# Thromboelastography with platelet mapping to guide anesthetic management of emergency cesarean delivery in a patient with thrombasthenia: a case report

**DOI:** 10.1186/s40981-023-00623-x

**Published:** 2023-05-25

**Authors:** Chieko Akinaga, Shingo Kawashima, Yuji Suzuki, Masako Matsumoto, Yoshiki Nakajima

**Affiliations:** 1 Department of Anesthesiology and Intensive Care Medicine, Hamamatsu University Hospital, 1-20-1 Handayama, Higashi-Ku, Hamamatsu-Shi, Shizuoka, 431-3192 Japan; 2 Department of Obstetrics and Gynecology, Hamamatsu University Hospital, Hamamatsu, Japan

**Keywords:** Thrombasthenia, Thromboelastography, Platelet function tests, Pregnancy, Cesarean section, Spinal anesthesia

## Abstract

**Background:**

Perinatal management of congenital platelet dysfunction represents a challenge. One of the major concerns is whether neuraxial anesthesia can be applicable for cesarean delivery. We present a patient with thrombasthenia who underwent emergency cesarean delivery.

**Case presentation:**

A 34-year-old primipara was diagnosed with autosomal dominant thrombasthenia, which was not classified as any known type. A thorough examination revealed that adenosine diphosphate aggregation and collagen aggregation were suppressed. Platelet mapping of viscoelastic testing was used to observe the trajectory of platelet function during pregnancy, which was found to be normal to hypercoagulable until 38 weeks of gestation. On the basis of the results of testing and physiological status, we commenced spinal anesthesia and avoided prophylactic platelet transfusion.

**Conclusion:**

The platelet mapping of viscoelastic testing was rapid and simple, allowing repeated examinations. We could choose the appropriate anesthesia method and determine the necessity of blood transfusion for a pregnant patient with thrombasthenia.

## Background

Congenital platelet dysfunction is a genetic disorder that causes difficulty in hemostasis and a tendency to bleed. The United Kingdom Haemophilia Centre Doctors’ Organization guideline describes that neuraxial anesthesia should be avoided in patients with Bernard Soulier syndrome or Glanzmann’s thrombasthenia, while it is allowed in patients with other congenital platelet function deficits only if the risks are outweighed by the benefits and the hemostatic defect has been corrected [1]. Although there are some reports about the management of pregnancy with Bernard Soulier syndrome or Glanzmann’s thrombasthenia [2–4], there is insufficient information on the anesthetic management of cesarean delivery for pregnant women with unclassified congenital platelet dysfunction.

Here, we describe our experience of the anesthetic management of an emergency cesarean delivery in a patient with congenital platelet dysfunction, which cannot be classified as a known disease. We continued to observe the trajectory of platelet function using Thromboelastography 6S Platelet Mapping (Haemonetics, Braintree, MA, USA) (TEGPM) and standard blood tests during pregnancy and the postpartum period.

Informed consent was obtained from the patient. This report adheres to the applicable EQUATOR guideline.

## Case report

A 34-year-old primiparous woman was referred to the obstetric anesthesia outpatient clinic at 26 weeks of pregnancy. The patient had histories of severe bruising in early childhood and difficulty in hemostasis after tooth extraction. She required blood transfusion during laparotomy for ovarian hemorrhage at the age of 15 years. Because of recurrent ovarian hemorrhage, her menstruation was controlled with estrogen and progesterone preparations. In addition, she had a family history of thrombocytopenia and intracranial hemorrhage. The patient’s platelet count hovered around 100,000/μL and was initially followed up as idiopathic thrombocytopenic purpura. At conization at age 25, 400 mL of platelet concentrate was transfused prophylactically, and the postoperative course continued without hemorrhagic complications.

A thorough examination at the age of 29 demonstrated normal ristocetin-induced platelet aggregation, suppressed adenosine diphosphate (ADP) aggregation to 39% when ADP 3.00 μM was added (normal range 70–90%), and collagen aggregation to 20% when collagen 2.00 μg/ml added (normal range 70–90%), but normal CD41 (glycoprotein IIb/IIIa) antigen, leading to a diagnosis of autosomal dominant, but not previously classified, thrombasthenia. After confirming the negative platelet antigen, she underwent fertility treatment at the age of 30.

To avoid unnecessary blood transfusions and provide a basis for selecting the anesthesia method when a cesarean section was necessary, we performed TEGPM as well as usual blood tests at 26, 32, 36, 38, and 39 weeks of gestation, and at 1 day, 5 days, 2 weeks, and 1 month after delivery. The results of the tests are shown in Tables 1 and 2. TEGPM results showed a normal to hypercoagulable state, and ADP aggregation was within the normal range until 38 weeks of gestation. The course of pregnancy was normal without obvious bleeding. The fetal growth remained within normal limits, and a vaginal delivery was planned.

**Table 1:** Laboratory test results

	During pregnancy					After delivery		
Parameter	26 weeks	32 weeks	36 weeks	38 weeks	39 weeks	Day 1	Day 5	2 weeks
Red blood cell count (10^4/^μL)	359	362	365	351	396	363	370	461
Hemoglobin concentration (g/dL)	11.4	11.2	11.0	10.2	11.2	10.1	10.2	12.4
Platelet count (10^4/^μL)	10.7	10.0	11.6	11.7	15.3	10.8	11.8	17.8
PT-INR	NA	NA	NA	NA	0.92	NA	0.86	NA
APTT (sec)	NA	NA	NA	NA	31.4	NA	29.6	NA
Fibrinogen concentration (mg/dL)	NA	NA	NA	NA	609	NA	521	NA

*Abbreviations: APTT* activated partial thromboplastin time, *NA* data are not available, *PT-INR* prothrombin time-international normalized ratio

**Table 2:** TEG platelet mapping results

	During pregnancy					After delivery			
Parameter (reference range)	26 weeks	32 weeks	36 weeks	38 weeks	39 weeks	Day 1	Day 5	2 weeks	1 month
R_HKH_ (min) (4.2–9.8)	4.5	4.1	5.3	4.8	3.7	3.4	3.7	4.8	5.6
K (min) (1.0–2.9)	1.5	1.3	1.2	1.2	0.8	0.8	1.0	1.3	2.0
Angle (deg) (57–75)	72.3	74.1	74.0	74.6	77.0	77.1	75.5	73.5	66.9
MA_HKH_ (mm) (53–68)	62.8	62.5	67.5	66.9	69.0	67.3	68.3	65.2	60.0
MA_ActF_ (mm) (2–19)	13.8	16.7	18.1	19.1	20.4	21.1	20.4	11.6	4.9
MA_ADP_ (mm) (45–69)	59.6	62.4	64.5	65.0	67.3	64.6	61.1	53.0	42.7
LY30 (%)	0.0	0.0	0.0	0.0	0.0	0.0	0.0	0.0	0.0
%Aggregation (ADP) (83–100)	93.5	99.8	93.9	96	96.5	94.2	85	77.2	68.6

*Abbreviations: ActF* activator F, *ADP* adenosine diphosphate, *HKH* heparinase kaolin, *K* coagulation time, *LY30* clot lysis at 30 min, *MA* maximum amplitude, *R* reaction time

The patient was admitted to the hospital with premature rupture of the membrane at 39 weeks of gestation. Two days after labor induction, an emergency cesarean section was performed with indications of labor arrest and nonreassuring fetal status. Because TEGPM results showed that ADP aggregation was within the normal range, blood coagulability had increased, and the patient did not manifest bleeding tendencies during pregnancy, we administered single-shot spinal anesthesia. The infant was admitted to the neonatal intensive care unit for transient tachypnea syndrome. The intraoperative blood loss was 480 g, and the postpartum blood loss was 210 g in the first 24 h after surgery. There were no postoperative complications and no prolongation of hospital stay. The baby was discharged from the neonatal intensive care unit on the fourth day after birth without bleeding tendency, and both mother and child were discharged on the sixth day. TEGPM revealed that ADP aggregation was not suppressed until delivery, although it continued to be suppressed after delivery and showed abnormal values 1 month later.

## Discussion

We measured platelet function using TEGPM during pregnancy and after delivery. Platelet function fluctuated during pregnancy but differed depending on the assay methods. ADP aggregation measured by light transmission aggregometry was suppressed before pregnancy, while it was within the normal range during pregnancy by TEGPM. The correlation between ADP aggregation values measured by these two methods is unclear during pregnancy. Light transmission aggregometry might have been able to be used during pregnancy, not only before pregnancy. However, it requires sample treatment and a specialist for precise measurement, which encouraged us to use TEGPM during pregnancy. We proceeded with spinal anesthesia without prophylactic platelet administration, which may lead to the development of antiplatelet antibodies. Maternal antiplatelet antibodies can cause fetal/neonatal alloimmune thrombocytopenia, leading to intrauterine fetal death and early postnatal intracranial hemorrhage [5]. In the present case, antiplatelet antibodies were negative before starting assisted reproductive technology, and there were no symptoms of maternal bleeding during the course of pregnancy with normal fetal growth and ultrasonographic findings, suggesting no fetal/neonatal alloimmune thrombocytopenia.

Monte [2] reported general anesthesia for emergency cesarean section in a pregnant woman with Glanzmann’s thrombasthenia. They administered platelet and recombinant factor VIIa under the monitoring of Thromboelastography. Wong [6] reported that no complications occurred in a case of thrombocytopenia caused by chronic portal hypertension after labor epidural analgesia, based on the results of thromboelastography. However, a consensus statement by The Society for Obstetric Anesthesia and Perinatology [7] does not recommend to use Thromboelastography or Rotational Thromboelastometry as a guide for neuraxial anesthesia in pregnant women with thrombocytopenia because of their limited association with clinical outcomes of acquired and congenital bleeding disorders, and of the very limited evidence to support their use before performing regional anesthesia in thrombocytopenic patients.

In the present case, however, the platelet count was within the normal range, and the type of platelet dysfunction was the suppression of ADP-induced aggregation. Thromboelastography is a simple and quick method to evaluate blood coagulability, including platelet function. TEGPM shows the degree of ADP aggregation and is useful for patients with apparent inhibition of ADP aggregation, as in this case. The pre-pregnancy values should have been used as the reference for management during pregnancy, but this was not possible because the patient was referred to us at 26 weeks of gestation. However, we obtained postpartum data and confirmed that ADP aggregation was inhibited at 1 month postpartum.

In conclusion, according to the results of TEGPM, ADP aggregation was normalized after 26 weeks of pregnancy in a patient with thrombasthenia, and it was useful in deciding the necessity of blood transfusion and whether regional anesthesia was an option.

## Abbreviations

Act F: Activator F
ADP: Adenosine diphosphate
APTT: Activated partial thromboplastin time
HKH: Heparinase kaolin
K: Coagulation time
LY30: Clot lysis at 30 min
MA: Maximum amplitude
PT-INR: Prothrombin time international normalized ratio
R: Reaction time
TEG-PM: Thromboelastography 6S Platelet Mapping
